# Lysosomal acid lipase in mesenchymal stem cell stimulation of tumor growth and metastasis

**DOI:** 10.18632/oncotarget.11244

**Published:** 2016-08-12

**Authors:** Ting Zhao, Cong Yan, Hong Du

**Affiliations:** ^1^ Department of Pathology and Laboratory Medicine, Indiana University School of Medicine, Indianapolis, IN, USA; ^2^ IU Simon Cancer Center, Indiana University School of Medicine, Indianapolis, IN, USA

**Keywords:** mesenchymal stem cell, lysosomal acid lipase, myeloid-derived suppressor cells, tumor growth and metastasis

## Abstract

Bone marrow mesenchymal stem cells (MSCs) are an important participant in the tumor microenvironment, in which they promote tumor growth and progression. Here we report for the first time that depletion of lysosomal acid lipase (LAL) in MSCs impairs their abilities to stimulate tumor growth and metastasis both in allogeneic and syngeneic mouse models. Reduced cell viability was observed in LAL-deficient (*lal*^−/−^) MSCs, which was a result of both increased apoptosis and decreased proliferation due to cell cycle arrest. The synthesis and secretion of cytokines and chemokines that are known to mediate MSCs' tumor-stimulating and immunosuppressive effects, i.e., IL-6, MCP-1 and IL-10, were down-regulated in *lal*^−/−^ MSCs. When tumor cells were treated with the conditioned medium from *lal*^−/−^ MSCs, decreased proliferation was observed, accompanied by reduced activation of oncogenic intracellular signaling molecules in tumor cells. Co-injection of *lal*^−/−^ MSCs and B16 melanoma cells into wild type mice not only induced CD8^+^ cytotoxic T cells, but also decreased accumulation of tumor-promoting Ly6G^+^CD11b^+^ myeloid-derived suppressor cells (MDSCs), which may synergistically contribute to the impairment of tumor progression. Furthermore, *lal*^−/−^ MSCs showed impaired differentiation towards tumor-associated fibroblasts. In addition, MDSCs facilitated MSC proliferation, which was mediated by MDSC-secreted cytokines and chemokines. Our results indicate that LAL plays a critical role in regulating MSCs' ability to stimulate tumor growth and metastasis, which provides a mechanistic basis for targeting LAL in MSCs to reduce the risk of cancer metastasis.

## INTRODUCTION

Tumor progression and metastasis are greatly influenced by non-malignant cells in the microenvironment. Many of these cells are derived from the bone marrow, and recruited by cancer cells to enhance their survival, growth, invasion and dissemination [1]. Mesenchymal stem cells (MSCs) are non-hematopoietic stem cells residing in the bone marrow that have the ability to self-renew and differentiate into multiple lineages, which contribute to tissue homeostasis and regeneration [2]. Recently, numerous studies have shown that bone marrow-derived MSCs participate in tumor progression by establishing a favorable tumor microenvironment [3–6]. MSCs not only support the tumor vasculature by differentiating into pericytes and perhaps endothelial cells, and secreting vasculogenic growth factors [6], but also differentiate into tumor-associated fibroblasts (TAFs), which establish cytokine networks that promote progression and migration [7]. In addition, MSCs display immunomodulatory properties by inhibiting proliferation and function of several major immune cells [8], which might be an important mechanism through which MSCs promote tumor growth or increase incidence of tumor formation.

Lysosomal acid lipase (LAL) is a key enzyme in the metabolic pathway of neutral lipids, which hydrolyzes cholesteryl esters and triglycerides in the lysosome of cells to generate free fatty acids and cholesterol. Genetic ablation of the *lal* gene in mice has profound pathogenic effects in multiple organs including myelopoiesis [9–11]. The effects of LAL deficiency on several types of cells, including aberrant growth and differentiation of myeloid lineage cells [11], impaired development and function of T cells [12], and abnormal proliferation and dysfunctions of endothelial cells [13] have been reported. However, the effect of LAL deficiency in MSCs is still unknown.

In the present study, the ability of LAL-deficient (*lal*^−/−^) MSCs in promoting tumor growth and metastasis was determined. The underlying mechanisms through which LAL deficiency affects MSC tumor-promoting functions were further investigated, including the effect of LAL deficiency on the MSC proliferation potential, synthesis and secretion of tumor-promoting cytokines and chemokines, *lal*^−/−^ MSCs-induced changes of immune cells, and MSCs' differentiation towards TAFs. In addition, the relationship between myeloid-derived suppressor cells (MDSCs) and MSCs was examined. Our study demonstrates that LAL deficiency in MSCs impairs their stimulation of tumor growth and metastasis by restricting MSC proliferation, reducing synthesis and secretion of tumor-promoting cytokines/chemokines, inducing CD8^+^ T lymphocytes and decreasing accumulation of MDSCs, and impairing differentiation towards TAFs. These findings provide a mechanistic insight into LAL in controlling MSCs' tumor-promoting functions. Melanoma B16 cell line and LLC lung cancer cell line were used for the investigation. Melanoma is a popular cancer form and a perfect model for easy detection of metastasis without extensive pathological dissection and characterization. Lung cancer is the leading cause of cancer deaths, accounting for approximately 27% of all cancer deaths.

## RESULTS

### LAL is required for MSCs′ stimulation of tumor cell growth and metastasis

**Figure 1:**
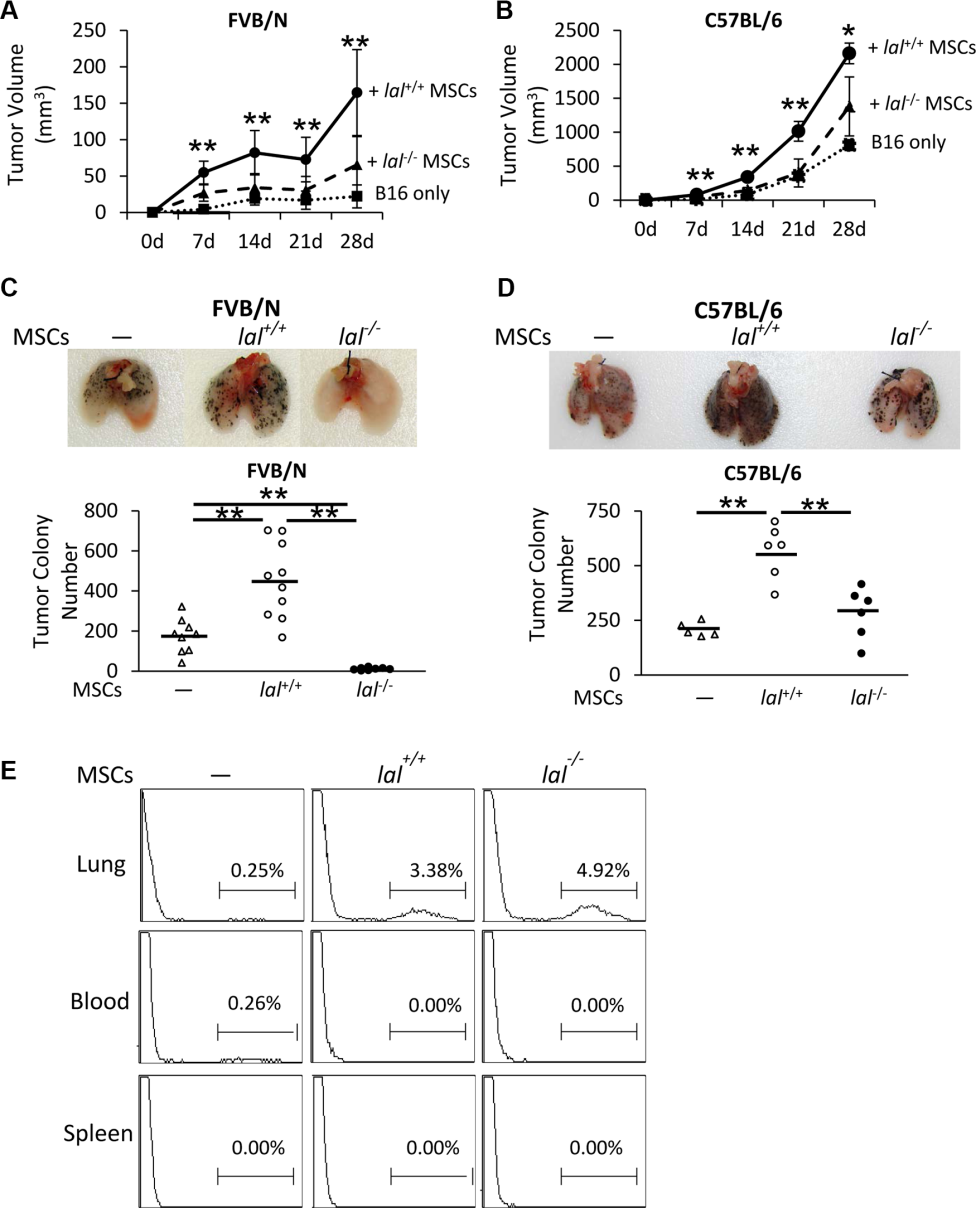
LAL is required for MSCs′ stimulation of tumor cell growth and metastasis (**A**) Statistical analysis of tumor volume (in cubic millimeters) at 7, 14, 21 and 28d post-injection in FVB/N mice. MSCs (6 × 10^5^) from *lal* ^+/+^ or *lal* ^−/−^ FVB/N mice and B16 melanoma cells (2 × 10^5^) were mixed, and then injected subcutaneously into the flank region of 3-month old *lal* ^+/+^ FVB/N mice. Data were expressed as mean ± SD; *n* = 6~10. **P < 0.01 vs. *lal* ^−/−^ MSCs. (**B**) Statistical analysis of tumor volume at 7, 14, 21 and 28d post-injection in C57BL/6 mice. The experimental condition was the same in (A). Data were expressed as mean ± SD; *n* = 3~6. *P < 0.05, **P < 0.01 vs. *lal* ^−/−^ MSCs. (**C**) Quantitative analysis of the melanoma colony numbers in the lungs of FVB/N mice. MSCs (8 × 10^5^) from *lal* ^+/+^ or *lal* ^−/−^ FVB/N mice and B16 melanoma cells (4 × 10^5^) were intravenously injected into *lal* ^+/+^ FVB/N mice for 2 weeks. Representative lungs are shown. Data were expressed as mean ± SD; *n* = 9 ~ 10. **P < 0.01. (**D**) Quantitative analysis of the melanoma colony numbers in the lungs of *lal* ^+/+^ C57BL/6 mice. The experimental condition was the same in (C). Representative lungs are shown. Data were expressed as mean ± SD; *n* = 5 ~ 6. *P < 0.05, **P < 0.01. (**E**) Flow cytometry analysis of MSCs in the lung, spleen and blood of FVB/N mice. CMFDA-labeled *lal* ^+/+^ or *lal* ^−/−^ FVB/N MSCs (1 × 10^6^) were intravenously co-injected with B16 melanoma cells (5 × 10^5^) into FVB/N *lal* ^+/+^ mice. Sixteen hours later, the mice were sacrificed and single cells from the lung, spleen and blood were harvested for flow cytometry analysis of the existence of MSCs. Representative pictures are shown. *n* = 4.

Numerous studies have shown that MSCs are involved in tumor progression and metastasis, during which they exert stimulatory effects [6]. To see whether LAL plays a role in regulating MSCs′ effect on tumor progression, the B16 melanoma cell model was used for subcutaneous and intravenous co-injection with bone marrow MSCs in both syngeneic C57BL/6 and allogeneic FVB/N mouse models. To examine the tumor growth potential *in vivo*, MSCs from C57BL/6 or FVB/N mice were mixed with B16 melanoma cells, and then co-injected subcutaneously into wild-type (*lal*^+/+^) C57BL/6 or FVB/N mice, respectively. B16 melanoma tumor grew faster in C57BL/6 syngeneic background than that in allogenic FVB/N background (Figure 1A and 1B). Compared with tumor growth in *lal*^+/+^ mice given only melanoma cells, both *lal*^+/+^ and *lal*^−/−^ MSCs facilitated tumor growth (Figure 1A and 1B). However, the tumors from *lal*^−/−^ MSCs-injected mice were significantly smaller than those developed in *lal*^+/+^ MSCs-injected mice at 7, 14, 21 and 28 days post-injection in both allogeneic and syngeneic conditions. Therefore, LAL deficiency in MSCs impaired their tumor growth stimulation effect.

Next, MSCs from C57BL/6 or FVB/N mice mixed with B16 melanoma cells were co-injected into the tail veins of *lal*^+/+^ C57BL/6 or FVB/N mice, respectively, to detect the metastatic potential. Two weeks after injection, compared to the *lal*^+/+^ C57BL/6 syngeneic mice that received melanoma cells only, the *lal*^+/+^ C57BL/6 mice co-injected with *lal*^+/+^ MSCs developed more melanoma colonies in the lungs (Figure 1D). In comparison, less B16 melanoma colonies were observed in the lungs of *lal*^+/+^ C57BL/6 syngeneic mice that received *lal*^−/−^ MSCs than those received *lal*^+/+^ MSCs under the syngeneic condition (Figure 1D). In allogeneic recipient FVB/N *lal*^+/+^ mice, melanoma metastasized less effectively due to immune rejection compared to syngeneic recipient C57BL/6 *lal*^+/+^ mice. Furthermore, co-injection of *lal*^−/−^ MSCs completely wiped out melanoma colonization in the lungs of *lal*^+/+^ FVB/N mice (Figure 1C). The above data suggested that LAL deficiency suppresses MSCs′ stimulation of tumor growth and metastasis. To further investigate whether MSCs exist in the metastatic sites, CMFDA-labeled MSCs from *lal^+/+^* and *lal^−/−^* mice were intravenously co-injected with B16 melanoma cells into the allogeneic FVB/N *lal*^+/+^ mice. Sixteen hours later, the mice were sacrificed and the lung, spleen and blood were harvested for flow cytometry analysis of the existence of MSCs. As shown in Figure 1E, labeled MSCs were found in the lungs of both *lal*^−/−^ MSC-injected and *lal*^+/+^ MSC-injected mice, but not in the spleen and blood. In consistent with this finding, no metastasis was observed in other organs within the investigated period.

### LAL is required for MSC proliferation

**Figure 2:**
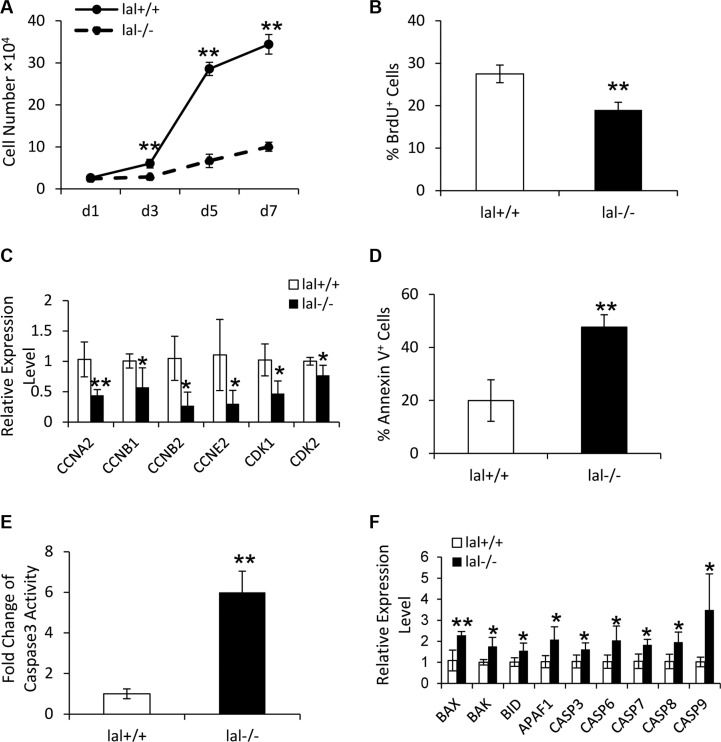
LAL is required for MSC proliferation (**A**) *lal* ^+/+^ and *lal* ^−/−^ MSCs growth at d1, d3, d5 and d7. MSCs were seeded at a density of 5 × 10^4^ cells/well in 6 well plates on day 0. On day 1, 3, 5, and 7, MSCs were trypsinized and counted with a hemocytometer. The cell growth curve was then established by a graphical analysis of the data. (**B**) The percentage of BrdU incorporation into *lal* ^+/+^ or *lal* ^−/−^ MSCs by flow cytometry. For both (A) and (B), data were expressed as mean ± SD; *n* = 5. **P < 0.01. (**C**) Real-time PCR analysis of mRNA expression levels of cell cycle-related molecules in *lal* ^+/+^ vs. *lal* ^−/−^ MSCs. (**D**) The percentage of Annexin V^+^ cells in *lal* ^+/+^ or *lal* ^−/−^ MSCs by flow cytometry analyses. Data were expressed as mean ± SD; n = 4. **P < 0.01. (**E**) The caspase 3 activity in *lal* ^+/+^ or *lal* ^−/−^ MSCs. Data were normalized to *lal* ^+/+^ MSCs and expressed as mean ± SD; *n* = 5. **P < 0.01. (**F**) Real-time PCR analysis of mRNA expression levels of apoptosis-related molecules in *lal* ^+/+^ vs. *lal* ^−/−^ MSCs. For both (C) and (F), the relative gene expression was normalized to GAPDH mRNA, and analysis was performed by the 2^−ΔΔCT^ method. Data were expressed as mean ± SD; *n* = 4. *P < 0.05, **P < 0.01.

LAL is known to influence cell proliferation in several cell types in *lal*^−/−^ mice [13, 14]. To assess the LAL influence on MSC proliferation, a cell growth curve assay was performed. As shown in Figure 2A, LAL deficiency decreased MSC proliferation in comparison to *lal*^+/+^ MSCs. This result was further confirmed by BrdU incorporation assay (Figure 2B). Consistent with growth arrest, LAL deficiency led to decreased expression of cell cycle-related genes, such as cyclin A (required for both S-phase progression and G2/M-phase transition), cyclin B (required for mitosis), cyclin E (required for G1/S-phase transition), and cyclin-dependent kinase (CDK) 1 and 2 (Figure 2C). The above data demonstrated that LAL is required for MSC proliferation.

The decreased cell viability in *lal*^−/−^ MSCs can occur as a result of either decreased proliferation, or increased apoptosis. Annexin V staining was performed to evaluate the apoptotic activity in *lal*^−/−^ vs. *lal*^+/+^ MSCs. As shown in Figure 2D, the percentage of Annexin V^+^ cells in *lal*^−/−^ MSCs was significantly greater than in *lal*^+/+^ MSCs, indicating increased apoptosis in *lal*^−/−^ MSCs. We further compared the caspase 3 activity between *lal*^+/+^ and *lal*^−/−^ MSCs. The result revealed that *lal*^−/−^ MSCs possessed higher caspase 3 activity than that of *lal*^+/+^ MSCs (Figure 2E), which was consistent with Annexin V staining. Accordingly, the expression of genes involved in pro-apoptosis was up-regulated in *lal*^−/−^ MSCs, including caspase 3, 6, 7, 8 and 9, BAX, BAK, BID, and APAF1 (Figure 2F). Taken together, these data suggested that LAL deficiency in MSCs restricts their proliferation by modulating cell cycle regulators and inducing cell death.

### LAL deficiency reduces synthesis and secretion of tumor-promoting cytokines and chemokines in MSCs

**Figure 3:**
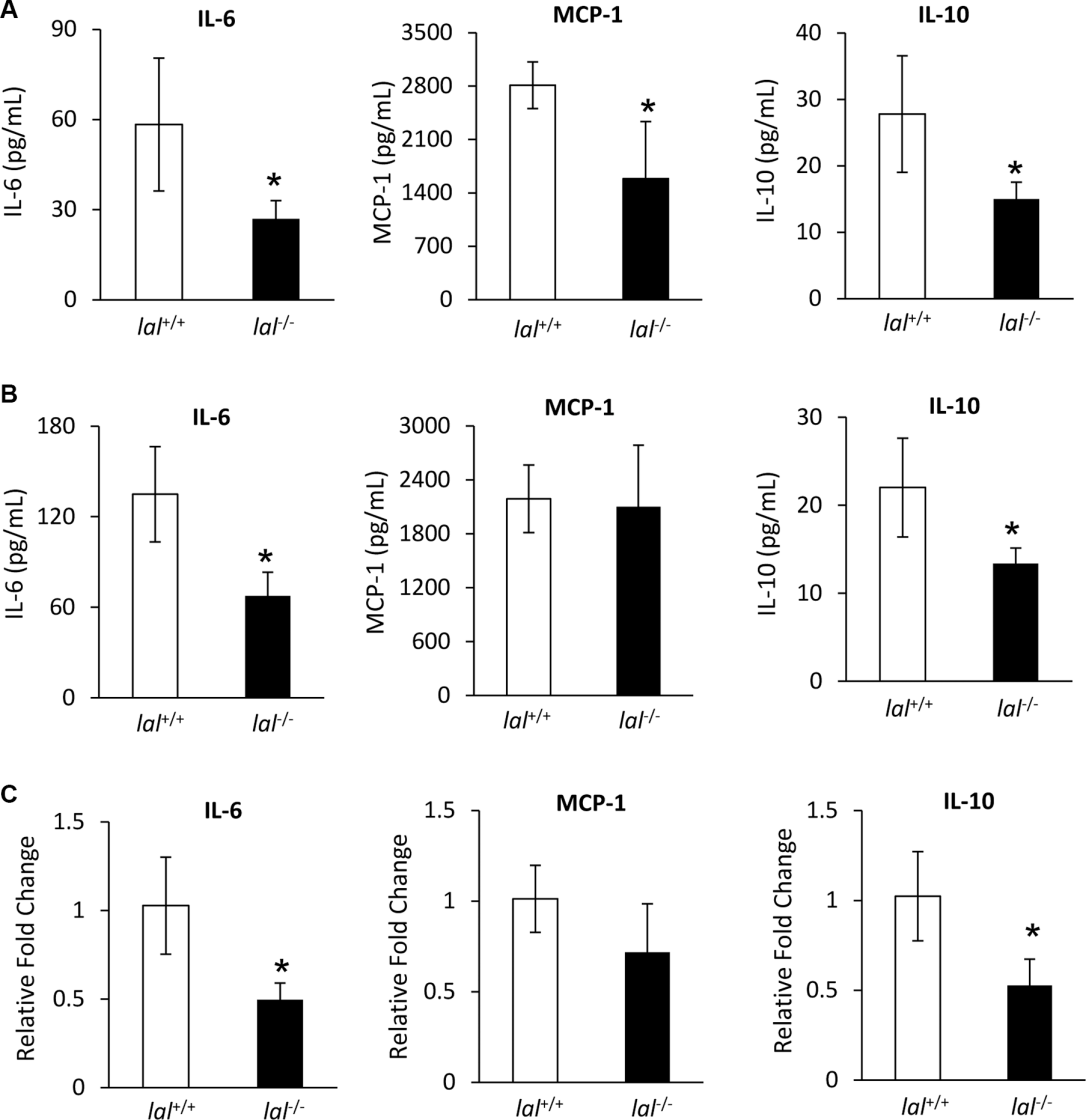
LAL deficiency reduced synthesis and secretion of tumor-promoting cytokines and chemokines in MSCs (**A**) The secretions of IL-6, MCP-1, and IL-10 in *lal* ^+/+^ or *lal* ^−/−^ FVB/N MSCs′ conditioned medium by ELISA analysis. MSCs were seeded at a density of 5 × 10^5^ cells/well in 6-well plates. Three days later, the conditioned medium was harvested for ELISA analysis. (**B**) The secretions of IL-6, MCP-1, and IL-10 of *lal* ^+/+^ or *lal* ^−/−^ C57BL/6 MSCs′ conditioned medium by ELISA analysis. For both (A) and (B), data were expressed as mean ± SD; *n* = 4. *P < 0.05. (**C**) Real-time PCR analysis of mRNA expression levels of IL-6, MCP-1, and IL-10 in *lal* ^+/+^ vs. *lal* ^−/−^ MSCs from FVB/N mice. The relative gene expression was normalized to GAPDH mRNA, and analysis was performed by the 2^−ΔΔCT^ method. Data were expressed as mean ± SD; *n* = 4. *P < 0.05.

MSCs have been reported to secrete a variety of cytokines and chemokines to influence tumor proliferation and migration [6]. In order to determine whether LAL deficiency in MSCs affects their secretion of tumor-facilitating cytokines/chemokines, conditioned medium (CM) of MSCs after 3-days culture was harvested. Then cytokines and chemokines that are known to promote tumorigenesis were measured by ELISA in MSCs isolated from both syngeneic and allogeneic mice. For MSCs derived from FVB/N mice, the concentrations of IL-6, MCP-1, and IL-10 were lower in *lal*^−/−^ MSC-CM compared with those in *lal*^+/+^ MSC-CM among tested cytokines and chemokines (Figure 3A). Similar results were observed in MSCs derived from C57BL/6 mice, except that there was no significant change in the MCP-1 level (Figure 3B). This observation has been confirmed at the mRNA level from FVB/N MSCs (Figure 3C). Therefore, reduced synthesis and secretion of IL-6, MCP-1, and IL-10 were, at least in part, responsible for the decreased stimulation of tumor growth and metastasis by *lal*^−/−^ MSCs, which formulate a unique signature for *lal*^−/−^ MSCs. We have also examined the levels of M-CSF, GM-CSF, G-CSF, IL-1β, TNFα and CCL-5 by ELISA, and VEGF and TGF-β by real-time PCR assay, which showed no significant changes (data not shown).

### MSC-CM stimulates proliferation of tumor cells by up-regulating activation of intracellular signaling molecules

**Figure 4:**
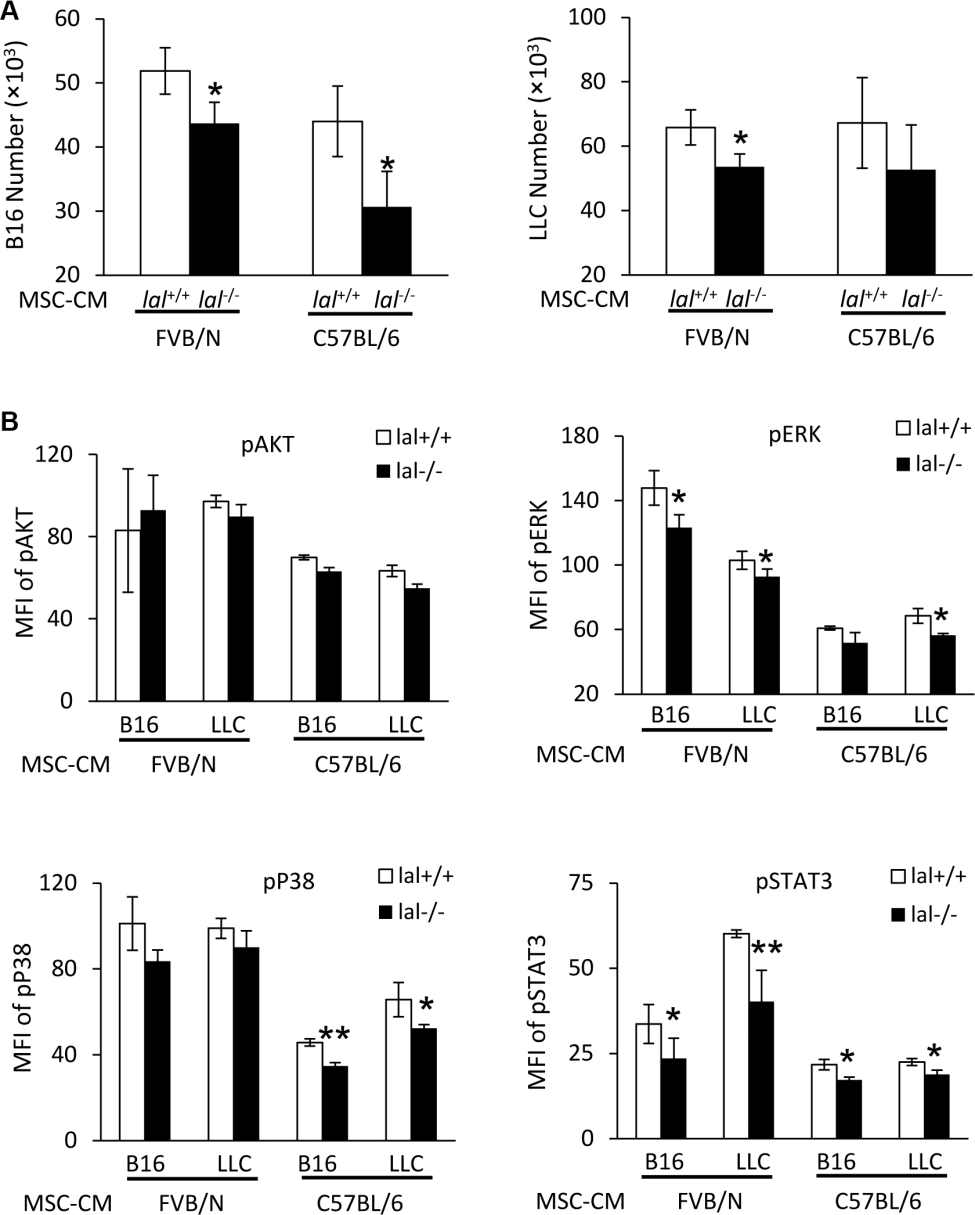
MSC-CM stimulates activation of intracellular signaling molecules (**A**) Stimulation of B16 melanoma cell proliferation by MSCs conditioned medium (CM). B16 melanoma cells (5 × 10^3^) or LLCs (1 × 10^4^) were seeded into 96-well plates, and then treated with CM from *lal* ^+/+^ or *lal* ^−/−^ FVB/N or C57BL/6 MSCs. The cell number was counted at 72 h after CM treatment. (**B**) Activation of intracellular signaling molecules in B16 melanoma cells by MSCs CM. Two hours after CM treatment, B16 melanoma or LLC cells were harvested for flow cytometry analysis. *lal* ^−/−^ MSC-CM decreased phosphorylation of ERK, P38 and STAT3 in B16 melanoma and LLC cells. Statistical analysis of mean fluorescent intensity (MFI) by flow cytometry is shown. In the above experiments, data were expressed as mean ± SD; *n* = 3~4. *P < 0.05, **P < 0.01.

To further confirm that MSC-CM is responsible for the decreased stimulation of tumor growth and metastasis by *lal*^−/−^ MSCs, tumor cells were treated with *lal*^+/+^ or *lal*^−/−^ MSC-CM, and their *in vitro* proliferation was examined. As shown in Figure 4A (left panel), compared with the *lal*^+/+^ MSC-CM treatment, both FVB/N and C57BL/6 *lal*^−/−^ MSC-CM showed a lower stimulatory effect on proliferation of B16 melanoma cells after 3 days. A similar defect was observed when *lal*^−/−^ MSC-CM was added to LLC cells (Figure 4A, right panel). To further investigate whether *lal*^−/−^ MSC-CM activates oncogenic intracellular signaling, a hallmark of tumor proliferation, tumor cells were treated with *lal*^+/+^ and *lal*^−/−^ MSC-CM, and intracellularly stained with anti-phospho-AKT, anti-phospho-ERK1/2, anti-phospho-NF-κB, anti-phospho-STAT3, or anti-phospho-P38 antibodies. Flow cytometry analyses revealed that the *lal*^−/−^ MSC-CM treatment showed lower activation of ERK1/2, STAT3, and P38 in B16 melanoma cells compared with that in *lal*^+/+^ MSC-CM-treated B16 cells in syngeneic C57BL/6 mice (Figure 4B). Similarly, activation of these oncogenic intracellular signaling molecules was also reduced in LLC cells with *lal*^−/−^ MSC-CM treatment (Figure 4B). The same observation was also made in allogeneic FVB/N mice, except activation of P38. We have reported that persistent activation of the STAT3 signaling pathway induced adenocarcinoma formation in the lung, and STAT3 serves as a biomarker for lung adenocarcinoma [15, 16]. Therefore, MSCs stimulate proliferation of tumor cells by CM through regulating activation of oncogenic intracellular signaling molecules.

### *lal*^−/−^ MSCs restore CD8^+^ T lymphocytes and decrease accumulation of Ly6G^+^CD11b^+^ MDSCs

**Figure 5:**
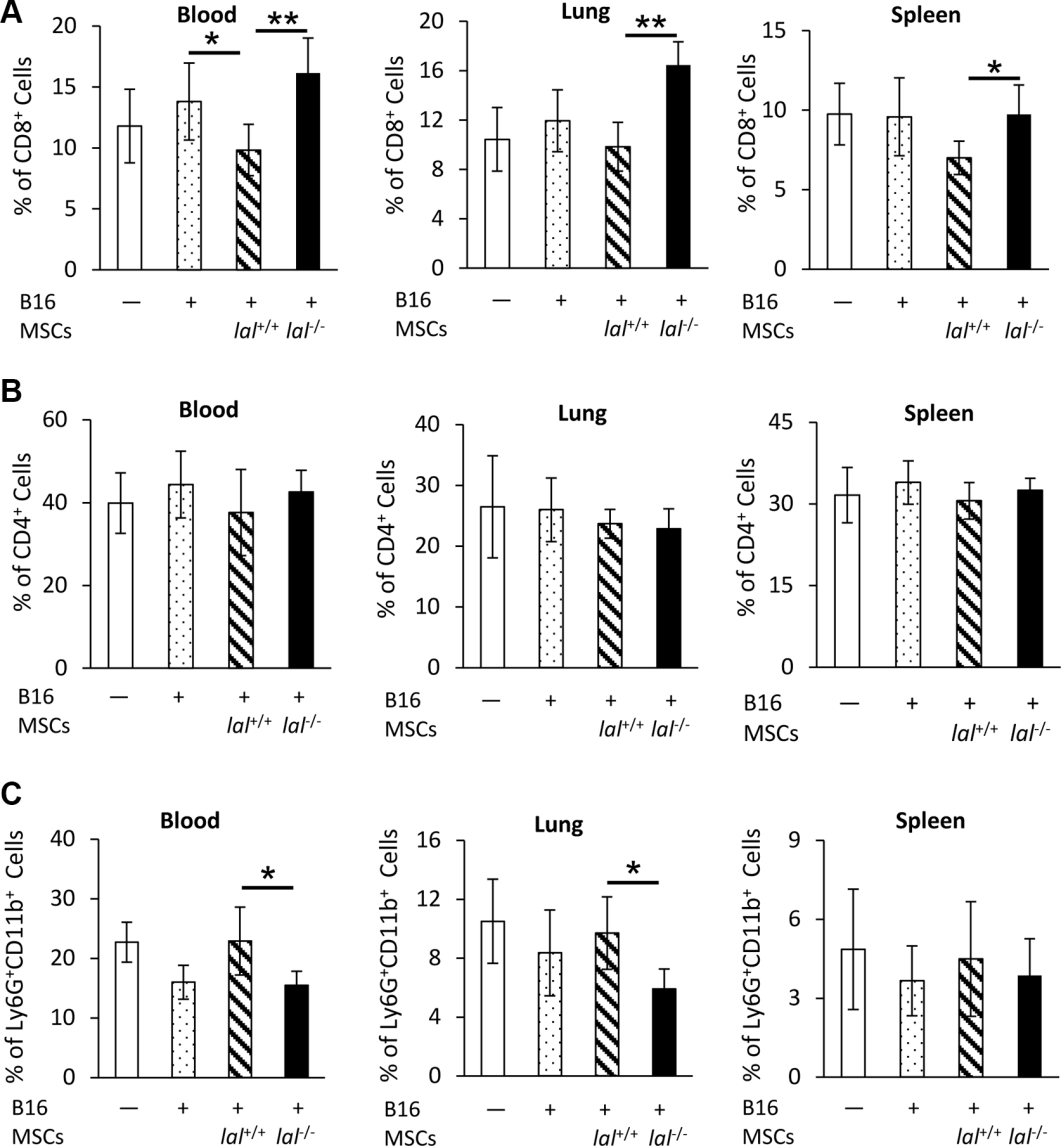
*lal* ^−/−^ MSCs restored CD8^+^ T lymphocytes and decreased accumulation of Ly6G^+^CD11b^+^ MDSCs B16 melanoma cells (4 × 10^5^) with or without MSCs (8 × 10^5^) from *lal* ^+/+^ or *lal* ^−/−^ FVB/N mice were intravenously co-injected into *lal* ^+/+^ FVB/N mice. Seven days later, the blood, lung and spleen were harvested for flow cytometry analysis of the percentage of CD8^+^ T cells (**A**), CD4^+^ T cells (**B**), and Ly6G^+^CD11b^+^ MDSCs (**C**). Data were expressed as mean ± SD; *n* = 5. *P < 0.05, **P < 0.01.

CD8^+^ T lymphocytes are a very important immune population involved in attacking tumor cells. MSCs have been reported to provide an immunosuppressive environment for tumor growth [17]. To investigate whether and how LAL deficiency influences MSCs′ ability in immunosuppression of T cells, MSCs and B16 melanoma cells were co-injected intravenously into *lal*^+/+^ FVB/N mice. The blood, lung and spleen were harvested 7 days later for flow cytometry analysis to determine the levels of CD4^+^ and CD8^+^ T cells. As Figure 5A demonstrated, mice injected with *lal*^+/+^ MSCs plus B16 melanoma cells systemically suppressed the CD8^+^ T cell population in the blood, lung and spleen. Mice injected with *lal*^−/−^ MSCs restored CD8^+^ T cell number back to the control level, or even higher. We did not observe the CD8^+^ T cell number difference between B16 tumor cell-injected and non-injected mice in these organs. No significant change in CD4^+^ T cells was observed in the blood, lung, and spleen of mice injected with *lal*^−/−^ vs. *lal*^+/+^ MSCs plus B16 melanoma cells (Figure 5B).

MDSCs are the well-known immune population that suppresses CD8^+^ T cells. Interestingly, the percentages of Ly6G^+^CD11b^+^ MDSCs were lower in the blood and lung of *lal*^−/−^ MSCs-injected mice, but not in the spleen compared with mice injected with *lal*^+/+^ MSCs (Figure 5C). These observations suggested that the impaired stimulation of tumor growth and metastasis by *lal*^−/−^ MSCs can be a result of restoration of the anti-tumor immunity.

### LAL deficiency impairs MSC differentiation towards TAFs

**Figure 6:**
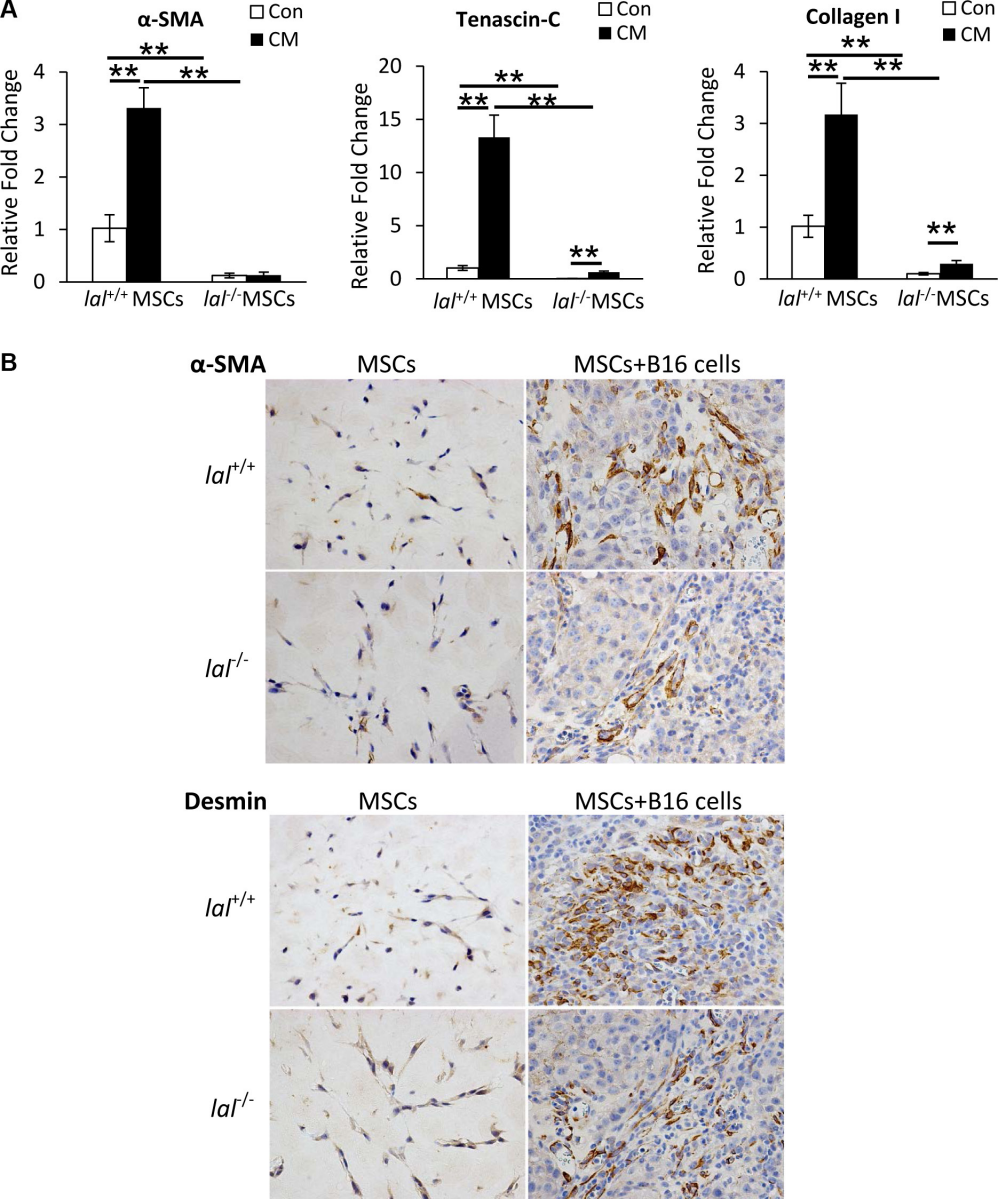
LAL deficiency impaired MSC differentiation towards (**A**) Real-time PCR analysis of mRNA expression levels of α-smooth muscle actin (α-SMA), tenascin-C (Tn-C), and collagen I in *lal* ^+/+^ or *lal* ^−/−^ MSCs treated with control medium (Con) or conditioned medium (CM) from B16 melanoma cells. MSCs were seeded at a density of 1 × 10^5^ cells/well into 6-well plates at day 0, and treated with 1 mL B16-CM or control medium. The medium was changed every 3 days. On day 14, MSCs were harvested for real-time PCR assay. The relative gene expression was normalized to GAPDH mRNA, and analysis was performed by the 2^−ΔΔCT^ method. Data were expressed as mean ± SD; *n* = 4. **P < 0.01. (**B**) Representative IHC staining of the matrigel plug sections using antibodies against α-SMA and desmin. MSCs (2 × 10^5^) from *lal* ^+/+^ or *lal* ^−/−^ FVB/N mice were mixed with or without B16 melanoma cells (1 × 10^5^) in matrigel, and the cell-matrigel-mixture was then injected subcutaneously into 3-month old *lal* ^+/+^ FVB/N mice. Fourteen days later, the mice were sacrificed, and the plugs were harvested for IHC staining. Original magnification, ×400.

TAFs have been reported to promote tumorigenesis in multiple tumor models, and MSCs have been found to express TAF antigens after exposure to the tumor microenvironment [6]. To see whether LAL deficiency influences MSCs′ differentiation towards TAFs *in vitro*, MSCs were treated with the conditioned medium (CM) of B16 melanoma cells for 2 weeks. The expression of TAF markers was then examined by real-time PCR assay. As Figure 6A demonstrated, B16 CM treatment up-regulated the gene expression of TAF markers α-smooth muscle actin (α-SMA), tenascin-C (Tn-C), and fibroblast marker collagen I in *lal*^+/+^ MSCs. Although the gene expression of Tn-C and collagen I was also increased in *lal*^−/−^ MSCs, their levels were significantly lower than those in *lal*^+/+^ MSCs, suggesting that LAL deficiency impaired MSCs′ differentiation into fibroblasts/myofibroblasts *in vitro*. For their *in vivo* differentiation, MSCs were mixed with B16 melanoma cells in matrigel, and then injected subcutaneously into *lal*^+/+^ mice. The matrigel plugs were harvested 2 weeks later for immunohistochemical staining against TAF markers, including α-SMA and desmin. In the plugs containing MSCs only, very few cells were positive for α-SMA or desmin, and there was no difference between *lal*^+/+^ and *lal*^−/−^ MSCs (Figure 6B). However, once the MSCs were within the tumor microenvironment, the expression of α-SMA and desmin was evident, with more positive cells in the plugs containing *lal*^+/+^ MSCs and B16 melanoma cells than those containing *lal*^−/−^ MSCs and B16 cells (Figure 6B). Taken together, LAL deficiency impairs MSC differentiation towards TAFs to support tumor growth.

### MDSCs partially restore decreased proliferation of *lal*^−/−^ MSCs

**Figure 7:**
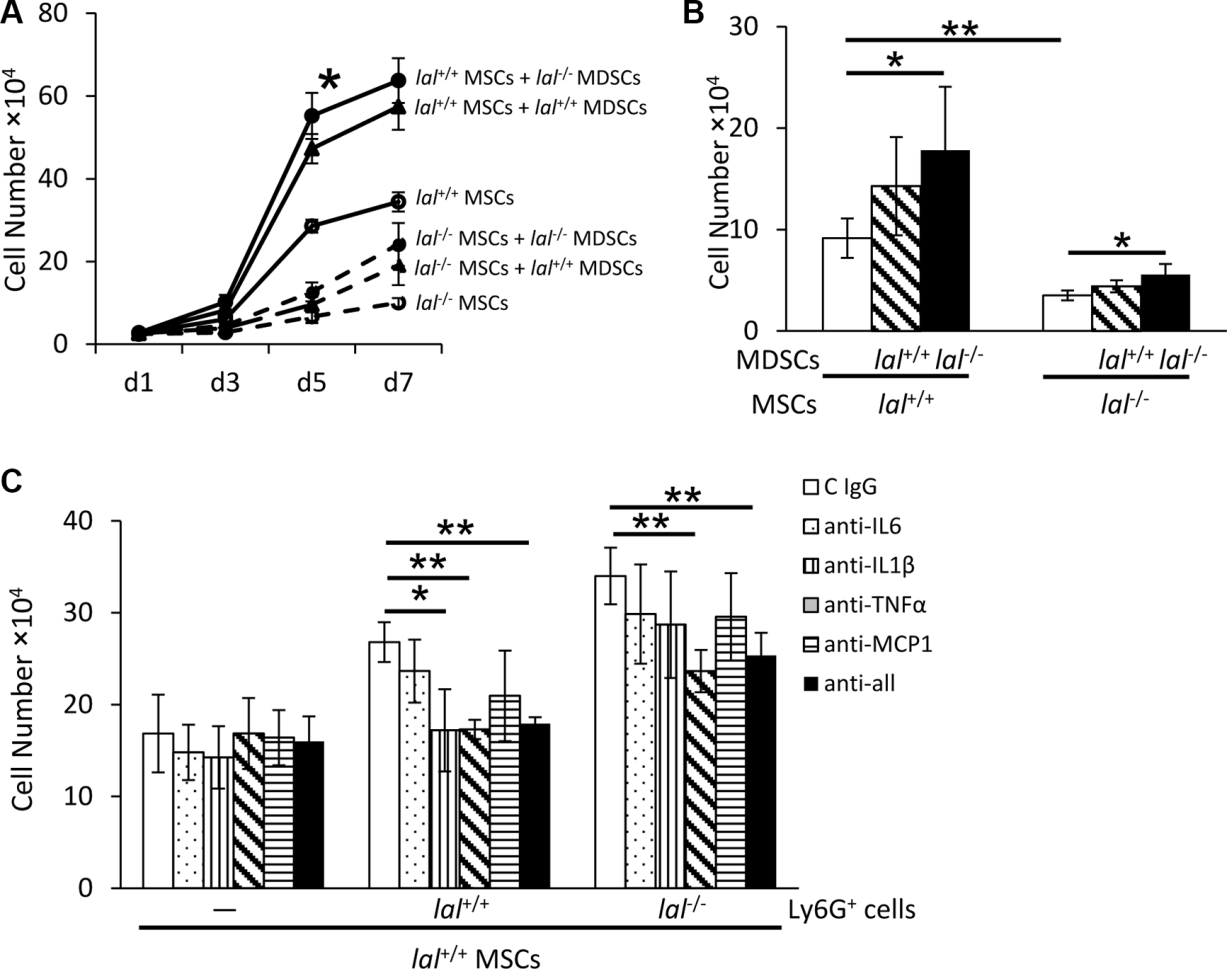
MDSCs partially restored decreased proliferation of *lal* ^−/−^ MSCs (**A**) *lal* ^−/−^ MDSCs stimulated MSC proliferation in the co-culture study. MSCs were seeded at a density of 5 × 10^4^ cells/well in 6-well plates, and *lal* ^+/+^ or *lal* ^−/−^ MDSCs (2 × 10^6^) were added to the well. The MSC number was counted at d1, d3, d5 and d7 afterwards. (**B**) *lal* ^−/−^ MDSCs stimulated MSC proliferation in the transwell study. MSCs (2 × 10^4^) were seeded into the lower chamber of transwells plates, while MDSCs (1 × 10^6^) were placed in the upper chamber. After 5 days, the MSC number was counted. (**C**) Neutralization of cytokines in MSC proliferation. *lal* ^+/+^ MSCs (2 × 10^4^) were seeded into the lower chamber of transwells, with MDSCs (1 × 10^6^) placed in the upper chamber. MDSCs were treated with 10 μg/ml neutralizing antibody against IL-6, IL-1β, TNF-α, MCP-1 individually, in combination or with control immunoglobulin G at day 1 and day 3. After 5 days, the number of MSCs was counted. In the above experiments, data were expressed as mean ± SD; *n* = 3~5. *P < 0.05, **P < 0.01.

In the bone marrow, the GR1^+^CD11b^+^ MDSCs population constitutes approximately 50% of total cells in *lal^+/+^* mice and 70% of total cells in *lal^−/−^* mice as we reported previously [11]. Considering that both MSCs and MDSCs originate from the bone marrow, it is necessary to examine the effect of MDSCs on MSC proliferation. MSCs were co-cultured with or without *lal*^+/+^ or *lal*^−/−^ MDSCs for 7 days. As shown in Figure 7A, MSC co-cultured with *lal*^−/−^ MDSCs showed increased proliferation than those with *lal*^+/+^ MDSCs. Even the decreased proliferation of *lal*^−/−^ MSCs was partially restored after co-culture with MDSCs, especially with *lal*^−/−^ MDSCs. *lal*^−/−^ MDSCs have been reported to exert their effects by secreting cytokines [13, 18]. To examine whether cytokines secreted by *lal*^−/−^ MDSCs facilitate MSC proliferation, a transwell study was performed with MDSCs seeding in the upper chamber and MSCs in the lower chamber. After 5 days' co-culture, the number of MSCs that were co-cultured with *lal*^−/−^ MDSCs was significantly increased (Figure 7B). When MDSCs were treated with anti-IL-6, IL-1β, TNFα or MCP-1 antibodies to neutralize cytokines in the transwell study, the stimulatory effect on MSCs proliferation was significantly inhibited by anti-TNFα antibody in both *lal*^+/+^ MDSCs and *lal*^−/−^ MDSCs groups (Figure 7C). Inhibition by IL-1β antibody was also observed in the *lal*^+/+^ MDSCs group, but not in the *lal*^−/−^ MDSCs group. Therefore, cytokines (especially TNFα) secreted by MDSCs were, at least in part, responsible for mediating stimulatory effects on MSC proliferation.

## DISCUSSION

MSCs display the ability to modulate the tumor microenvironment, thus having an impact on tumor growth, progression and metastasis. Here we used B16 melanoma cells as a model to compare the tumor-promoting ability between *lal*^+/+^ and *lal*^−/−^ MSCs, and to investigate the underlying mechanisms involved in the process. The present study shows for the first time that MSCs with LAL deficiency lose the ability to stimulate tumor growth and metastasis.

It has been reported that B16 melanoma cells did not form tumors in allogeneic mice unless MSCs were co-injected [17]. In our case, although small tumors were observed in allogeneic FVB/N mice with B16 melanoma cell injection, when B16 melanoma cells were mixed with *lal*^+/+^ MSCs, their ability to grow and migrate was significantly enhanced (Figure 1A and 1C). However, LAL deficiency greatly reduced this ability of MSCs in the allogeneic FVB/N mouse model. It is interesting that *lal*^−/−^ MSCs almost completely inhibited B16 metastasis (Figure 1C).

Many mechanisms have been proposed to account for MSC-mediated effects of tumor support [6]. During the process of MSC-mediated tumor growth, MSCs need to maintain certain cell viability to perform their functions. As we reported before, LAL plays differential roles to determine the cell fate in different cell types. LAL deficiency promotes proliferation and inhibits apoptosis of myeloid lineage cells and endothelial cells [13, 19]. In an opposite effect, LAL deficiency inhibits proliferation and induces apoptosis of T lymphocytes [12]. Similar to T lymphocytes, here we report that the decreased cell viability of *lal*^−/−^ MSCs was observed during *in vitro* culture experiments. Concomitantly, a higher caspase 3 activity was observed in *lal*^−/−^ MSCs than that in *lal*^+/+^ MSCs, accompanied by up-regulated expression of the caspase family members and the pro-apoptotic genes (Figure 2). Klopp et al. suggested that the increased tumor mass observed when MSCs and tumor cells were co-injected can be related to the increased proliferation of MSCs in tumors [6], indicating that MSC proliferation is critical to tumor growth. Funes et al. reported that restoration of Nrf2 function in transformed MSCs impaired *in vivo* tumor growth by mechanisms that included MSC sensitization to apoptosis [20], suggesting that MSC apoptosis is one of the reasons for the impaired tumor growth. Therefore, the decreased proliferation and increased apoptosis provide a mechanism by which *lal*^−/−^ MSCs lose their viability and ability to stimulate tumor growth and metastasis.

Other mechanisms also play important roles in MSCs to stimulate tumor growth. MSCs secrete a variety of growth factors, cytokines and chemokines to influence tumor proliferation, migration, and angiogenesis [6]. Reduced synthesis and secretion of IL-6, MCP-1 and IL- 10 were observed in *lal*^−/−^ MSCs (Figure 3). MSC-secreted IL-6 has been found to act as a paracrine factor to sustain breast cancer cell migration [21]. Besides its effects on tumor cells, MSCs themselves are also the target of IL-6. Under the influence of IL-6, MSCs can transform malignant cells and have tumorigenic properties [22]. In addition, IL-6 maintains MSC stemness by enhancing proliferation and protecting from apoptosis. Such stemness-maintaining effects by IL-6 support our findings of the decreased proliferation and increased apoptosis in *lal*^−/−^ MSCs (Figure 2). MCP-1 was reported to facilitate breast-tumor metastasis by recruiting inflammatory monocytes [23]. IL-10 permits malignant cells escaping from cell-mediated immune defenses, and is associated with poor prognosis in colon cancer [24]. Moreover, IL-6, MCP-1 and IL-10 are related to the immunoregulatory effects of MSCs [25]. When B16 melanoma or LLC cells were treated with conditioned medium from *lal*^−/−^ MSCs, decreased proliferation of tumor cells was observed (Figure 4A), accompanied by reduced activation of ERK1/2, p38MAPK, and STAT3 oncogenic molecules in tumor cells (Figure 4B and 4C). This is in agreement with a previous report that MSC-secreted IL-6 facilitates cancer cell migration by persistent activation of MAPK, AKT and p38MAPK in breast cancer cells [21]. It is also agreed with our previous report that persistent activation of STAT3 induces adenocarcinoma in the lung, and STAT3 serves as a biomarker for lung adenocarcinoma in humans [15, 16]. It is well known that the downstream metabolic derivatives of LAL serve as ligands for PPARγ, which controls expression of IL-6 [26]. We have shown that LAL downstream derivative 9-HODE treatment significantly reversed pathogenesis in *lal^−/−^* mice, including tumor growth and tumor invasion [27, 28]. In addition, we have reported that metabolic enzyme LAL influences gene transcription of AKT, mTOR and STAT3 [14, 29], which control the secretion of MCP-1 and IL-10 and IL-6 [30, 31].

MSCs possess immunosuppressive effects, which serves as another important mechanism through which MSCs promote tumor growth and progression. Djouad et al. reported that the immunosuppressive function of MSCs led to a higher incidence of melanoma formation in a mouse model [17]. MSCs can directly inhibit proliferation and impair the function of a variety of immune cells, such as dendritic cells, T and B lymphocytes, and natural killer cells [6]. When MSCs and B16 melanoma cells were co-injected into wild type mice, there was no difference of CD4^+^ T cells between *lal*^+/+^ and *lal*^−/−^ MSC-injected mice (Figure 5B). However, the percentage of CD8^+^ T cells was increased in the blood, lung and spleen of *lal*^−/−^MSC-injected mice compared with *lal*^+/+^ MSC-injected mice (Figure 5A). Interestingly, the percentage of T cell-suppressing and tumor-promoting Ly6G^+^CD11b^+^ MDSCs was decreased in the blood and lung of *lal*^−/−^ MSC-injected mice (Figure 5C). It is conceivable that the synergistic effect of increased CD8^+^ cytotoxic T cells and decreased MDSCs induced by *lal*^−/−^ MSCs contributes to impaired tumor growth and metastasis. Conversely, *lal*^−/−^ MDSCs facilitated MSC proliferation through MDSC-secreted cytokines and chemokines (Figure 7).

Fibroblasts are an important component of stroma cells in the tumor microenvironment that support tumor growth. MSCs have the capacity to differentiate into TAFs within the tumor microenvironment, which is another potential mechanism underlying MSCs′ stimulation of tumor growth and progression. TAFs are characterized by the presence of several markers. After exposure to the tumor microenvironment, MSCs expressed TAF markers including Tn-C, α-SMA, desmin, and fibroblast marker Collagen I (Figure 6), indicating their differentiation towards TAFs. *lal*^−/−^ MSCs exhibited lower levels of these TAF markers than those of *lal*^+/+^ MSCs (Figure 6), serving as another mechanism to explain why LAL deficiency impairs MSCs′ tumor-stimulating ability.

In conclusion, LAL deficiency in MSCs results in the loss of tumor-promoting functions through regulating cell viability, secretion of paracrine factors, immunosuppressive function and fibroblastic differentiation. The LAL metabolic pathway can be a novel target to modulate the tumor-promoting functions of MSCs and reduce the risk of cancer progression and metastasis.

## MATERIALS AND METHODS

### Animals and cell lines

Wild-type (*lal*^+/+^) and *lal*^−/−^ mice of the FVB/N and C57BL/6 background were bred in house. All scientific protocols involving the use of animals have been approved by the Institutional Animal Care and Use Committee of Indiana University School of Medicine and followed guidelines established by the Panel on Euthanasia of the American Veterinary Medical Association. Animals were housed under Institutional Animal Care and Use Committee-approved conditions in a secured animal facility at Indiana University School of Medicine.

The murine B16 melanoma cell line and Lewis lung carcinoma (LLC) cell line (ATCC, Manassas, VA, USA) were cultured in DMEM supplemented with 10% FBS (Gibco, Grand Island, NY, USA).

### MSC isolation

MSCs were isolated from the bone marrow of femurs and tibias of 8-week-old FVB/N or C57BL/6 mice and cultured in MSC basal medium supplemented with MSC stimulatory supplements (STEMCELL Technologies, Vancouver, Canada) as previously described [32]. Cells were used at low passages P2 or P3 to avoid senescence-associated effects progressively acquired with passage [33].

### Cell growth curve

MSCs were seeded at a density of 5 × 10^4^ cells per well in a 6 well plate on day 0. On day 1, 3, 5, and 7, MSCs were trypsinized and counted with a hemocytometer. The cell growth curve was then established by a graphical analysis of the data.

### BrdU incorporation

BrdU incorporation was performed using the BrdU Flow Kit (BD Biosciences, San Jose, CA, USA) as we previously described [11]. Briefly, BrdU was added to MSCs at a final concentration of 10 μmol/L. One hour later, cells were collected and fixed. After permeabilization, cells were incubated with DNase I at 37°C for 1 h, followed by labeling with anti-BrdU antibody for 20 min at room temperature. Cells were then analyzed by flow cytometry.

### Real-time RT-PCR

Total RNAs from MSCs were purified using the Qiagen total RNA purification kit (Qiagen, Valencia, CA, USA). Quantitative (q)RT-PCR was performed as described previously [34]. Analysis was performed by the 2^−ΔΔCT^ method. Primers of mIL-6, mMCP-1, mIL-10, mCCNA2, mCCNB1, mCCNB2, mCCNE2, mCDK1, mCDK2, mBAX, mBAK, mBID, mAPAF1, mCaspase 3, 6, 7, 8 and 9, α-SMA, Tn-C, collagen I A1, and GAPDH for real-time PCR were described previously [12, 34–36].

### Annexin V staining

Dual staining with FITC–annexin V and propidium iodide (PI) was performed to detect cells undergoing apoptosis using an annexin V–FITC kit (BD Biosciences) as we described previously [12]. MSCs were harvested, and resuspended in annexin V-binding buffer containing FITC-conjugated annexin V. PI was then added into cells and incubated on ice for 10 min. Nonspecific binding was blocked by pre-incubating cells with rat IgG (10 mg/mL) and anti-FcII/III. Cells were analyzed on a LSRII machine (Becton Dickinson, Franklin Lakes, NJ, USA) within 1 h. Viable cells were defined by FITC^−^ and PI^−^ population. Early apoptotic cells were defined by FITC^+^ and PI^−^ population.

### Caspase 3 activity assay

Caspase 3 activity in MSCs was detected using Caspase-3 Assay Kit according to the manufacturer's instructions (BD Biosciences). Briefly, cells were lysed in cold cell lysis buffer for 30 min on ice. The cell lysate was then incubated with caspase-3 substrate Ac-DEVD-AMC at 37°C for 1 h. The caspase-3 activity was quantified using a spectrofluorometer with excitation at 380 nm and emission at 440 nm.

### Mouse tumor growth and metastasis model

The tumor growth and metastasis model have been described recently [18]. MSCs and B16 melanoma cells were collected separately. A pilot study has been performed to determine the best ratio between MSCs and B16 melanoma cells. To test the tumor growth potential, 6 × 10^5^ MSCs isolated from FVB/N or C57BL/6 mice and 2 × 10^5^ B16 melanoma cells were mixed, centrifuged and re-suspended in 100 μL PBS, and then injected subcutaneously into the flank region of 3-month old recipient *lal*^+/+^ mice in the FVB/N or C57BL/6 background. Tumor volume (length × width^2^ × π/6) was monitored every week for 4 weeks. To test the metastasis potential, 8 × 10^5^ MSCs and 4 × 10^5^ B16 melanoma cells were mixed, centrifuged and re-suspended in 200 μL PBS, and then injected intravenously into 3-month old *lal*^+/+^ FVB/N or C57BL/6 mice. Two weeks after the injection, the mice were sacrificed and the lungs were inflated with 4% paraformaldehyde for examination of metastasis.

### Isolation of bone marrow-derived MDSCs

MDSCs were isolated as we previously described [18, 29]. As previously published, almost all *lal^−/−^* MDSCs are Ly6G^+^Ly6C^+^, and almost all *lal^−/−^* MDSCs are CD11b^+^Ly6G^+^ cells [18, 29]. Therefore, to simplify the isolation procedure, Ly6G antibody-coupled magnetic beads were used and sufficiently isolate *lal^−/−^* MDSCs from the *lal^−/−^* bone marrow, and the equivalent control from the wild type bone marrow [11, 37]. Briefly, bone marrow cells were isolated from the femurs and tibias of mice. Cells were first incubated with biotin-conjugated anti-Ly6G antibody at 4°C for 15 min. After washed with PBS, cells were incubated with anti-biotin microbeads at 4°C for another 15 min. Subsequently, cells were subjected to magnetic bead sorting according to the manufacturer's instructions (Miltenyi Biotec., Auburn, CA, USA).

### *In vitro* co-culture of MSCs and MDSCs

A pilot study was performed to determine the best ratio between MSCs and MDSCs. MSCs (5 × 10^4^) and MDSCs (2 × 10^6^) were mixed, and seeded into a well of 6-well plates at day 0. At day 1, 3, 5, and 7, unattached MDSCs were removed by washing with PBS, and the number of attached MSCs was counted. Morphologically, MDSCs are much smaller than MSCs, making them easily excluded.

### Flow cytometry analysis

For measurement of intracellular signaling molecules in B16 melanoma and LLC cells, 2 h after MSC-conditioned medium (CM) treatment, cells were harvested, fixed and permeabilized using BD Cytofix/Cytoperm Fixation/Permeabilization Kit (BD Biosciences) according to the manufacturer's instructions, and then incubated with Alexa Fluor 647-conjugated anti-phospho-Akt antibody, anti-phospho-P44/42 (Erk1/2) antibody, anti-phospho-NF-κB antibody, anti-phospho-P38 antibody, and anti-phospho-Stat3 antibody (Cell Signaling Technology, Beverly, MA, USA) at 4°C overnight. In the following day, cells were washed and ready for flow cytometry analysis.

For immune cell profile analysis in mice that were co-injected with MSCs and B16 melanoma cells, single cells from the blood, spleen and lung were harvested as previously described [12, 38]. Cells were labeled with anti-Ly6G, anti-CD11b, anti-CD4, and anti-CD8 cell surface antibodies (eBioscience, San Diego, CA, USA) at 4°C for 15 min, and then washed for flow cytometry analysis.

For analysis of MSC existence in the metastatic sites, 1 × 10^6^ CMFDA-labeled *lal*^+/+^ or *lal*^−/−^ FVB/N MSCs were intravenously co-injected with B16 melanoma cells (5 × 10^5^) into FVB/N *lal*^+/+^ mice. Sixteen hours later, the mice were sacrificed, and single cells from the lung, spleen and blood were harvested as previously described for flow cytometry analysis of MSC existence [12, 38]. For flow cytometry analysis, ≥10,000 cells were acquired and scored using a LSRII machine (BD Biosciences). Data were processed using the CellQuest software program (BD Biosciences).

### Cytokine measurement by ELISA

MSCs were seeded at a density of 5 × 10^5^ cells per well in 6-well plates. Three days later, the conditioned medium was harvested. The expression levels of IL-6, IL-10, and monocyte chemoattractant protein (MCP) −1 (BD Biosciences) in the conditioned medium were measured using ELISA kits according to the manufacturer's instructions.

### MSC differentiation towards TAFs

For *in vitro* differentiation, *lal*^+/+^ or *lal*^−/−^ MSCs were treated with control medium or conditioned medium (CM) from B16 melanoma cells for 14 days. B16 melanoma cells were grown in DMEM +10% FBS culture medium and CM was harvested after 24 h. CM was centrifuged and supernatant was collected. MSCs were seeded at a density of 1 × 10^5^ cells/well of 6-well plates, and treated with 1 mL B16-CM or DMEM +10% FBS control medium at day 0. The medium was changed every 3 days. On day 14, MSCs were harvested for real-time PCR assay of α-SMA, Tn-C and collagen I.

For *in vivo* differentiation, 2 × 10^5^ MSCs from *lal*^+/+^ or *lal*^−/−^ FVB/N mice were mixed with or without B16 melanoma cells (1 × 10^5^) in matrigel, and the cell-matrigel-mixture was then injected subcutaneously into 3-month old *lal*^+/+^ FVB/N mice. Fourteen days later, the mice were sacrificed, and the plugs were harvested for IHC staining against TAF marker α-SMA and desmin by Pathology Laboratory of Indiana University School of Medicine.

### Transwell assay

To observe the effect of MDSCs-secreted cytokines on MSC proliferation, transwell assay was performed with 0.4-μm-pore 6.5-mm diameter Transwell plates (Corning, Corning, NY, USA) to separate MDSCs and MSCs. One million *lal*^+/+^ or *lal*^−/−^ MDSCs in 200 μL media were seeded into the upper chamber of the plates, while 2 × 10^4^ MSCs in 500 μL media were placed in the lower chamber. For the neutralization study, MDSCs were treated with 10 μg/mL neutralizing antibody against IL-6, IL-1β, TNF-α, MCP-1 or control IgG. After 5 days' culture, the transwells were removed, and the number of MSCs in the lower chamber was counted.

### Statistics

Data were expressed as mean ± SD. Differences between two treatment groups were compared by Student's *t*-test. When more than two groups were compared, one-way ANOVA with post-hoc Newman-Keul's multiple comparison test was used. Results were considered statistically significant when *P* < 0.05. All analyses were performed with GraphPad Prism 5.0 (GraphPad, San Diego, CA, USA).
